# Near-infrared spectroscopy monitoring during immediate transition after birth: time to obtain cerebral tissue oxygenation

**DOI:** 10.1007/s10877-017-0052-9

**Published:** 2017-08-19

**Authors:** Evelyn Ziehenberger, Berndt Urlesberger, Corinna Binder-Heschl, Bernhard Schwaberger, Nariae Baik-Schneditz, Gerhard Pichler

**Affiliations:** 1 Research Unit of Neonatal Micro- and Macrocirculation, Department of Paediatrics, Medical University of Graz, Graz, Austria; 2 Division of Neonatology, Department of Paediatrics, Medical University of Graz, Auenbruggerplatz 34, 8036 Graz, Austria

**Keywords:** Near-infrared-spectroscopy, Neonate, Immediate transition, Time periods

## Abstract

Feasibility of cerebral tissue oxygenation measurements immediately after birth has been published starting with first values 2 min after birth. Aim of this study was to evaluate, the time periods from birth and from arrival at the resuscitation table to obtain the first cerebral tissue oxygenation values with two different near infrared spectroscopy (NIRS) devices. The present study is an analysis of exploratory parameters of two prospective observational studies. Cerebral tissue oxygen saturation was measured by the NIRO 200NX measuring “cerebral-tissue-oxygenation-index” (cTOI) or the INVOS5100C measuring “cerebral-regional-oxygen-saturation” (crSO_2_). Four time periods (T) were defined: T1 birth to arrival at resuscitation table, T2 arrival to application of NIRS sensor, T3 application to first displayed cTOI or crSO_2_ value, and T4 from arrival at resuscitation table to first displayed values. Additionally, we compared first displayed values of cTOI and crSO_2_. Thirty neonates were included. Twenty-four were term and six late-preterm neonates. Fifteen neonates measured with NIRO were compared to 15 measured with INVOS. T1 was 49 (6–163) s with NIRO versus 59 (15–87) s with INVOS, T2 14 (4–20) s versus 12 (15–18) s, T3 33 (13–138) s versus 17 (6–290) s and T4 46 (20–153) s and 34 (14–300) s. The first displayed value tended to be higher for cTOI [54% (18–80)] compared to crSO_2_ [35% (15–87)]. There were no significant differences between devices in time periods and first values displayed. Cerebral tissue oxygenation can be measured within 1 min after arriving at the resuscitation table in term and preterm neonates after birth without difference between devices.

## Introduction

During immediate transition after birth beside the clinical assessment of newly born infants, routine monitoring with pulse oximetry/ECG is recommended. These two non-invasive continuous monitoring methods enable measurement of the arterial oxygen saturation (SpO_2_) and heart rate. Unfortunately, these methods do not provide information about potentially compromised oxygen delivery to the brain. Therefore, there is a growing interest, to monitor the cerebral regional tissue oxygenation by using near-infrared spectroscopy (NIRS) during immediate postnatal transition [1–3]. There are different NIRS devices available, but two most widely used devices in neonates are the NIRO 200 NX (Hamamatsu Photonics, Japan) providing “tissue oxygenation index” (TOI) and the INVOS 5100C (Covidien, USA) providing “regional tissue oxygen saturation” (rSO_2_). With both NIRS devices centiles of cerebral tissue oxygenation immediately after birth starting with first values 2 min after birth have been already published [4, 5]. For an efficient medical monitoring during crucial situations, such as the immediate transition period of newborn infants after birth, monitoring devices should be easily applicable and the first values have to be provided quickly. Several studies already reported on time periods to obtain first SpO_2_ values with pulse oximetry immediately after birth [6–8]. These studies showed that it is possible to obtain first SpO_2_ values in less than 1 min [9]. However, there is no study which has investigated the time needed to apply a NIRS sensor and to obtain first TOI/rSO_2_ values after the arrival of a newborn infant at the resuscitation table. Therefore, the aim of this study was to evaluate, the time periods from birth and from arrival at the resuscitation table to obtain the first cerebral tissue oxygenation values with two different devices. We hypothesized that there is no difference between these two devices.

## Methods

The present study was a post-hoc analysis of exploratory parameters of different prospective observational studies performed at the Medical University of Graz (Regional Committee on Biomedical Research Ethics approval No. 23-403 ex 10/11, 25-342 ex 12/13). Parental informed consent was obtained from all individual participants included in the study. Videos of term and preterm infants during immediate transition after birth were analysed, which were recorded during these prospective studies. The video started when the whole body was delivered. Immediately after birth, the neonates were brought by the midwife from the operating theatre and placed on the resuscitation table under an overhead heater. The forehead skin area was cleaned and dried.

In neonates either the NIRS sensor of the NIRO 200 NX (Hamamatsu Photonics, Japan) was positioned on the right forehead to evaluate the “cerebral tissue oxygenation index” (cTOI) or the neonatal sensor of INVOS 5100C (Covidien, USA) on the left forehead for evaluating the “cerebral regional oxygen saturation” (crSO_2_). Neonates were excluded when the arrival-time or application of sensor was not clearly visible on video recordings or if both NIRS sensors were applied simultaneously in one neonate. For analyses neonates, in whom INVOS 5100C was applied, were matched for gestational age (±1 week) to neonates in whom NIRO 200 NX was applied. Neonates with application of both sensors simultaneously were excluded. The sensor was fixed in both groups on the forehead with a double adhesive plaster and/or gauze bandage by a research team member, who was not involved into resuscitation. Four time periods (T) were defined: T1 from birth to arrival of the neonate on the resuscitation table, T2 from arrival to application of the NIRS sensor, T3 from application to first cTOI or crSO_2_ values on the monitor, and T4 from arrival to first displayed NIRS values. Additionally, we analysed first displayed values of cTOI and crSO_2_.

The data are presented as mean ± standard-deviation (±SD) for normally distributed continuous data and median (range) when the distribution was skewed. Data of NIRO group and INVOS group were compared using paired t-test and Wilcoxon-rank-sum-test for parametric and non-parametric continuous variables, respectively, and chi square or Fisher’s exact test for categorical variables. A p value of 0.05 was considered significant.

## Results

**Fig. 1:**
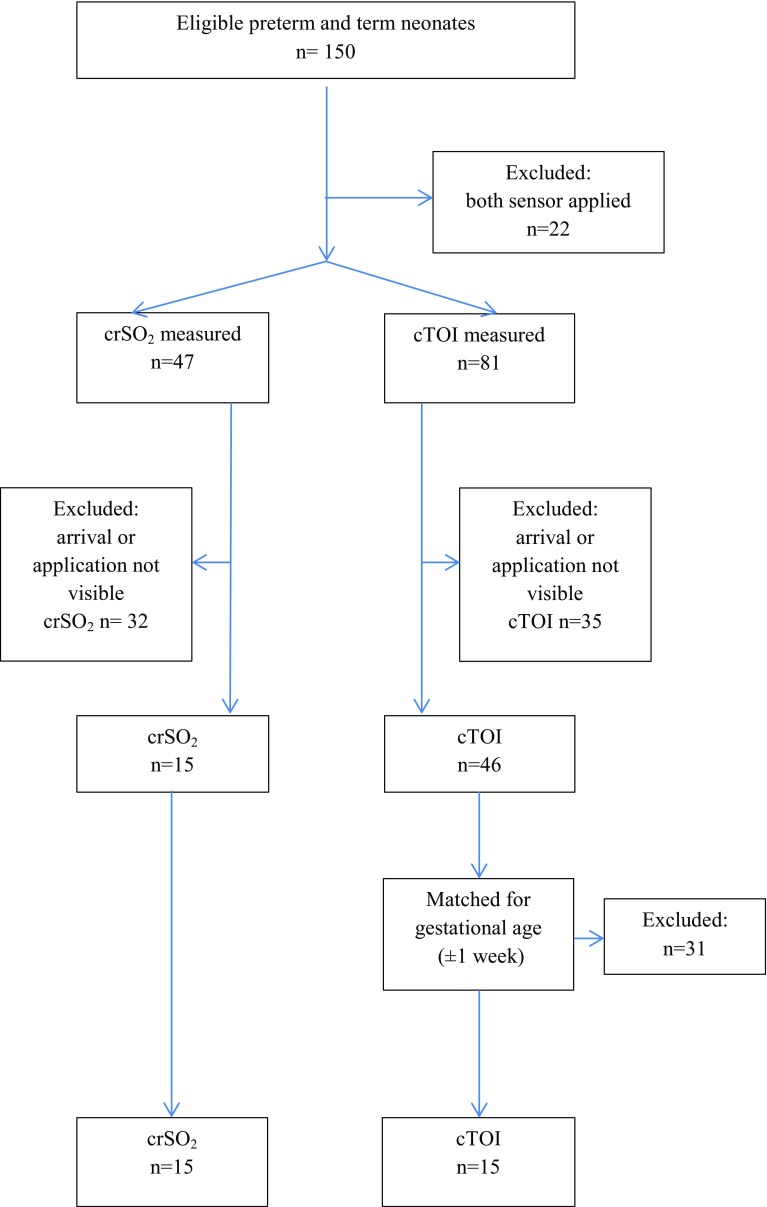
Flow chart of included and excluded term and preterm neonates with NIRS monitoring during immediate transition

Out of the 150 neonates with NIRS measurements during the first 15 min after birth, 15 neonates measured with INVOS 5100C were included, in whom time of arrival and application of the sensors were clearly visible. Main exclusion criterion was hindered visibility caused by attending staff (Fig. 1). These neonates were matched for gestational age to 15 neonates in whom the NIRO 200 NX sensor was applied (Fig. 1). Of the 15 neonates from the NIRO 200 NX cohort 7 were male and four were preterm neonates. Out of the 15 neonates from the INVOS 5100C cohort six were male and two were preterm neonates. The demographic data of the study population is presented in Table 1. The results of the four defined time periods are presented in Table 2. The median time periods from arrival of the neonate on the resuscitation table and first value displayed tended to be shorter with INVOS 5100C compared to NIRO NX 200 without reaching significance. Shortest time periods were within 20 s with NIRO 200 as well as with INVOS 5100C. First displayed values of cTOI tended to be higher compared to crSO_2_, also without reaching significance.

**Table 1:** Demographic data of 30 term and preterm neonates

	NIRO, n = 15	INVOS, n = 15	p value
Gestational age (weeks)	38 ± 2	38 ± 3	0.51
Birth weight (g)	3125 ± 645	3198 ± 434	0.74
Apgar 1 min	9 (6–10)	9 (5–9)	0.87
Apgar 5 min	10 (9–10)	10 (8–10)	0.77
Apgar 5 min	10 (9–10)	10 (9–10)	0.58
Female (n)	8	9	0.60
Term neonates >37 (n)	11	13	0.65
Gestational age (weeks)	39 ± 1	39 ± 1	0.59
Birth weight (g)	3212 ± 507	3314 ± 328	0.25
Late preterm neonates <37 (n)	4	2	0.65
Gestational age (weeks)	36 ± 1	35 ± 1	0.85
Birth weight (g)	2888 ± 992	2440 ± 170	0.91

p value <0.05

**Table 2:** Time periods of the NIRO 200NX and INVOS 5100C

	NIRO n = 15	INVOS n = 15	p value
Birth to arrival at the resuscitation table (s)	49 (6–163)	59 (15–87)	0.96
Arrival to application of NIRS transducer (s)	14 (4–30)	12 (5–18)	0.30
Application to first value (s)	33 (13–138)	17 (6–290)	0.58
Arrival to first displayed value (s)	46 (20–153)	34 (14–300)	0.64
Initial cTOI or crSO_2_ value (%)	54 (18–80)	35 (15–87)	0.19

p value <0.05

## Discussion

The present study demonstrates that after the arrival of the neonate on the resuscitation table non-invasive cerebral NIRS monitoring is feasible within 1 min. The application of the NIRS sensor on the neonate’s forehead can be performed quickly independently of which device was used enabling first values even within 20 s after arrival of the neonate at the resuscitation table. However, most studies present data of cerebral regional tissue oxygenation starting 2 min after birth [3–5]. The main reason for this delay, as in the present study, is the delay in arrival of the neonate at the resuscitation table after birth. Present time periods of application to first NIRS signal after arrival of the neonate at the resuscitation table are comparable with time periods observed to obtain first signals with pulse oximetry [7, 9]. For SpO_2_ using the Nellcor OxiMax it has been demonstrated that it took 22–32 s [6], using the Masimo Radical 7 pulse oximeter 79 s [7] and using the Masimo Radical 7 pulse oximeter with two different applications it took 10–16 s [8]. However, in the present study in some neonates it took several minutes to obtain first signals. This was mainly due to dislocation of sensors and manipulation. Same problems are well known when pulse-oximetry is used [7, 9]. Both, SpO_2_ and NIRS monitoring require careful technical and practical preparations to obtain the initial values within 1 min [9]. The signal quality of NIRS can especially be improved with careful application, since it is influenced by vernix, moisture, bruise, oedema or dense growth of hair [10]. In the present study time periods to obtain the first displayed signal was similar with both devices. However, the first displayed value tended to be lower with the INVOS 5100C device using the neonatal sensor compared to the NIRO 200 NX. This difference is well known especially in the lower regions of tissue oxygenation [11] and published centiles with both devices [4, 5].

## Conclusion

Cerebral tissue oxygenation can be measured within 1 min after arriving at the resuscitation table in term and preterm neonates after birth without difference between devices. Therefore, NIRS has the potential to become an additional routinely used tool for cerebral monitoring during immediate transition and resuscitation after birth.
